# Bypassing the guardian: regulated cell death pathways in p53-mutant cancers

**DOI:** 10.1186/s11658-025-00751-5

**Published:** 2025-06-14

**Authors:** Jonathan Y. Chung, Bruce A. Knutson

**Affiliations:** Department of Biochemistry and Molecular Biology, State University of New York Upstate Medical University, Syracuse, NY 13210 USA

**Keywords:** P53, Cancer, Metastasis, Apoptosis, Necroptosis, Ferroptosis, Reactive oxygen species

## Abstract

Approximately half of all cancers bear mutations in the tumor suppressor p53. Despite decades of research studying p53 function, treatment of p53-mutant cancers remains challenging owing to the effects of p53 mutations on many complex and interrelated signaling networks that promote tumor metastasis and chemoresistance. Mutations in p53 promote tumor survival by dysregulating cellular homeostasis and preventing activation of regulated cell death (RCD) pathways, which normally promote organismal health by eliminating dysregulated cells. Activation of RCD is a hallmark of effective cancer therapies, and p53-mutant cancers may be particularly susceptible to activation of certain RCD pathways. In this review, we discuss four RCD pathways that are the targets of emerging cancer therapeutics to treat p53-mutant cancers. These RCD pathways include E2F1-dependent apoptosis, necroptosis, mitochondrial permeability transition-driven necrosis, and ferroptosis. We discuss mechanisms of RCD activation, effects of p53 mutation on RCD activation, and current pharmaceutical strategies for RCD activation in p53-mutant cancers.

**Graphical Abstract:**

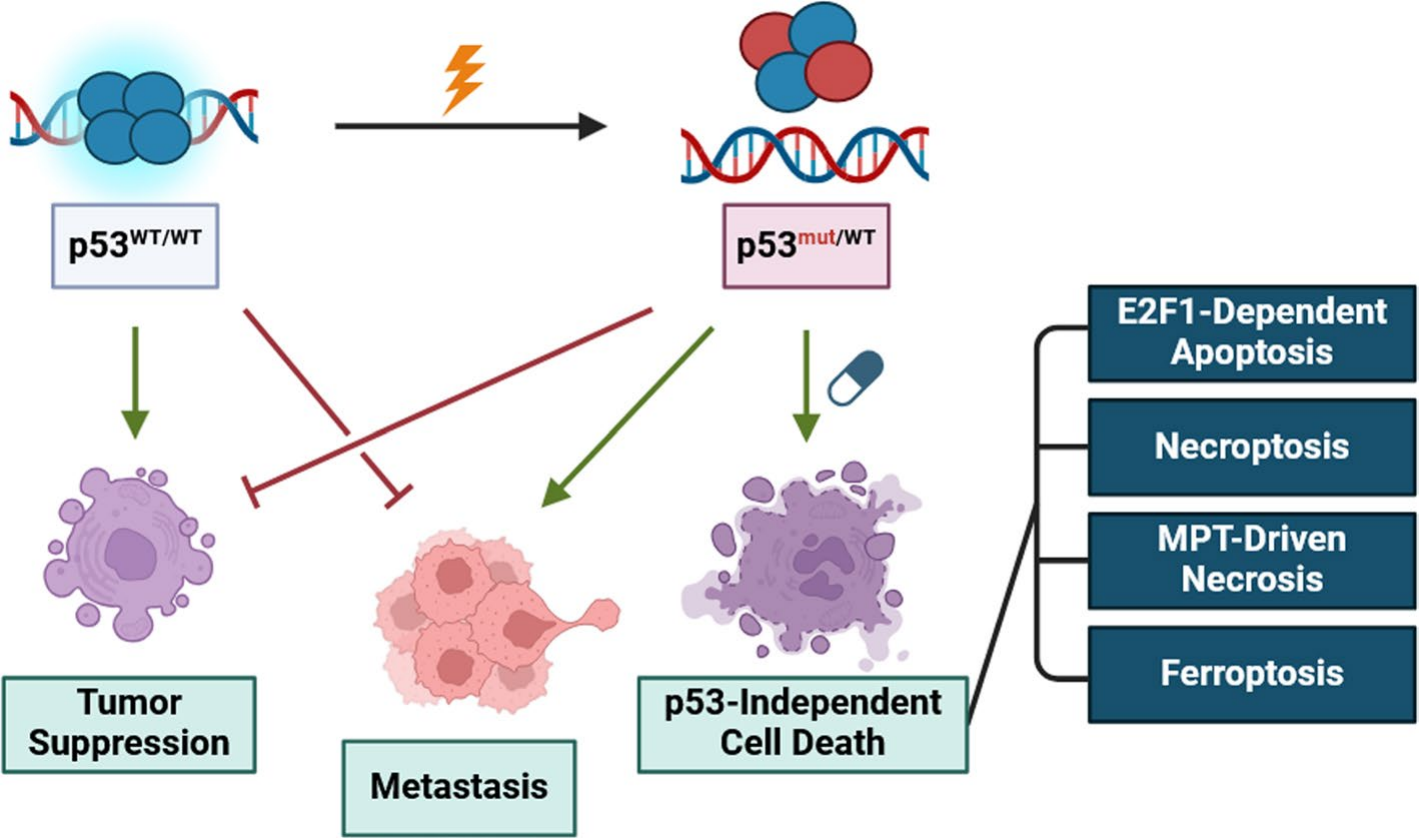

## Introduction

Biomedical scientists have extensively studied the p53 transcription factor since David Lane and Lionel Crawford discovered p53 in 1979 [1]. After being misidentified as an oncoprotein, p53’s important function in tumor suppression was only appreciated after establishment of the murine wild-type p53 protein sequence [2]. A total of 10 years after the initial discovery of p53, Bert Vogelstein and colleagues demonstrated that p53 mutation and deletion occur at an extremely high frequency in colorectal carcinomas, providing compelling evidence that p53 functions as a tumor suppressor [3]. Generations of researchers have since shown that p53, known as the “guardian of the genome,” mediates several critical functions in tumor suppression including DNA damage repair, metastasis prevention, and induction of growth arrest and apoptosis.

**Fig. 1:**
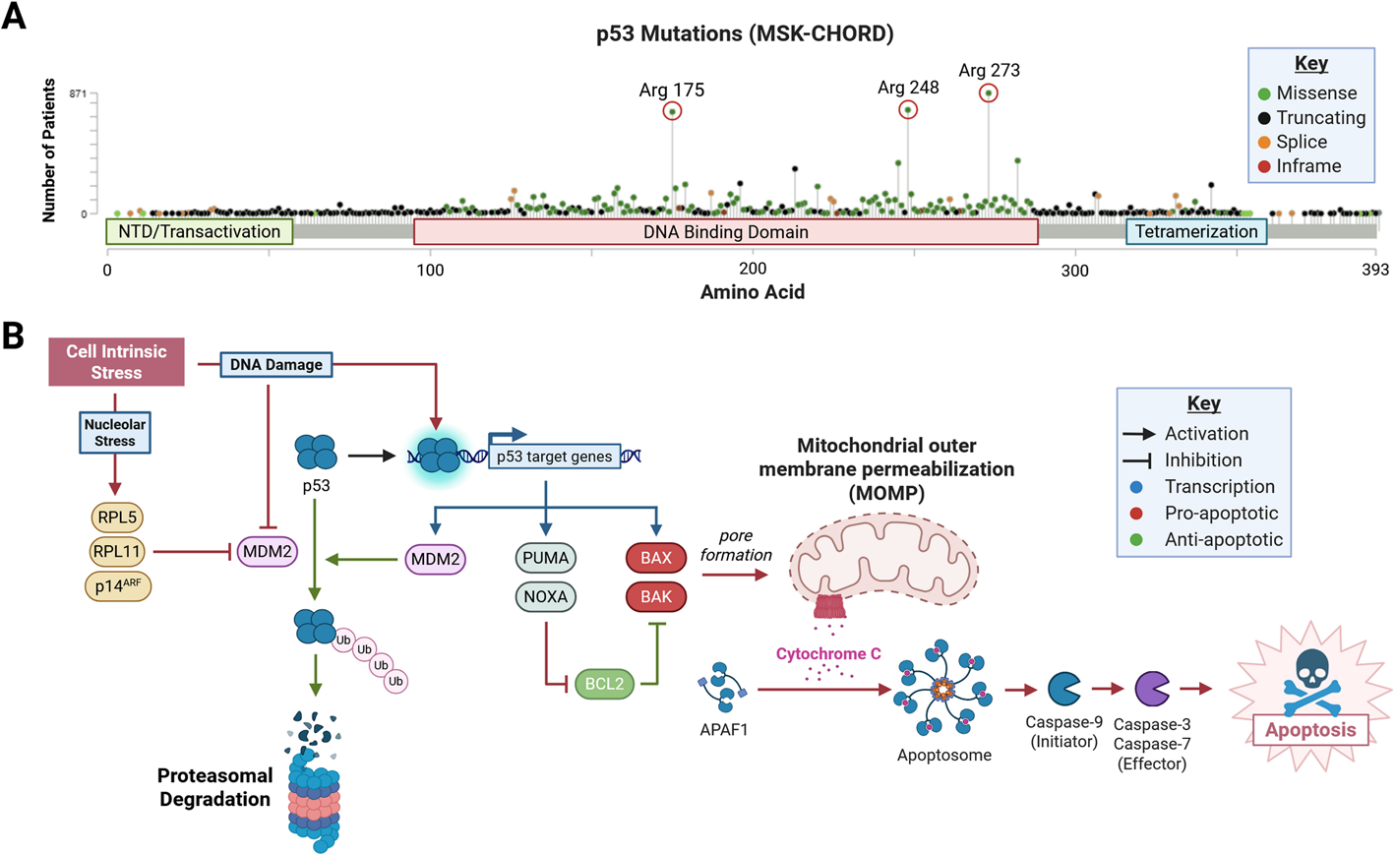
**p53**-dependent intrinsic apoptosis. **A** Lollipop plot of p53 mutations generated with cBioPortal (MSK-CHORD) [267–270]. p53 mutations primarily occur in the DNA binding domain and are enriched in hotspots (red circles). **B** Activation of p53 by cell intrinsic stresses such as DNA damage and nucleolar stress leads to p53-dependent apoptosis. Intrinsic stresses lead to MDM2 inhibition, resulting in p53 stabilization. Further posttranslational modification of p53 results in transcription of proapoptotic genes. BAX and BAK proteins form pores in the outer mitochondrial membrane, leading to leakage of cytochrome c from the mitochondrial intermembrane space. Cytochrome c oligomerizes with cytosolic APAF-1, forming apoptosome protein complexes that activate caspases, resulting in widespread proteolysis and cell death. Created in BioRender. Chung, J. (2025) https://BioRender.com/08778hb

p53’s role in apoptosis has garnered particular interest with respect to cancer therapy. Apoptosis and other regulated cell death (RCD) pathways maintain organismal homeostasis by removing unneeded or unwanted cells [4]. In tumor surveillance, activation of RCD pathways prevents progression of premalignant cells to cancers as tumorigenic characteristics arise. RCD pathways hold special relevance to cancer therapy as many treatments including conventional chemotherapies and radiation therapy trigger tumor apoptosis [5, 6]. p53 promotes *intrinsic apoptosis* by transcriptionally activating proapoptotic factors in response to cell intrinsic stress such as DNA damage and nucleolar stress (Fig. 1B). In unstressed tissues, the E3 ubiquitin ligase mouse double minute 2 (MDM2) maintains p53 at low levels by facilitating p53 proteasomal degradation [7, 8]. However, when cell-intrinsic stress increases, MDM2 is inhibited, allowing p53 to accumulate [9, 10]. Stress also triggers p53 posttranslational modifications, such as phosphorylation and acetylation, which enhance p53 transcriptional activity [11]. Provided sufficient stimulus, p53 activates an apoptotic program that leads to transcription of p53-upregulated modulator of apoptosis (*PUMA*/*BBC3*), and phorbol-12-myristate-13-acetate-induced protein 1 (*NOXA*/*PMAIP1*), which promote pore formation on the mitochondrial outer membrane by BCL2-associated X protein (BAX) and BCL2 antagonist/killer 1 (BAK) [12]. Cytochrome c may then escape the mitochondrial intermembrane space and form the apoptosome by oligomerizing with cytosolic apoptotic peptidase activating factor 1 (APAF-1) [13]. Intrinsic apoptosis culminates with activation of caspase proteases by apoptosomes, leading to widespread proteolysis, DNA fragmentation, and cell death.

Similar to with other tumor suppressors, cancers disrupt p53 function to promote tumorigenesis. p53 is the most frequently mutated gene in cancers [14], and p53 mutation predicts poor prognosis across several cancer types [15]. Importantly, p53 mutations are highly diverse, and mutant p53 function is dependent on the specific mutation present. Due to the diversity of p53 mutations and their differential effects on p53’s behavior, efforts have been made to categorize p53 mutations into functional classes. For example, p53 mutations have been divided into either disruptive or nondisruptive mutations based on their location and charge characteristics [16, 17]. Poeta et al. found that disruptive p53 mutations, defined as resulting in an early stop codon or an alteration in the critical DNA binding domain of p53, were associated with worse survival in head and neck squamous cell carcinoma [16]. Certain disruptive p53 mutations are highly enriched in cancer, affecting hotspots including arginine 175 and arginine 273 in p53’s DNA binding domain (Fig. 1A) [18]. Generally, disruptive p53 mutations disturb p53’s normal function; however, several specific mutations may also confer novel tumorigenic properties to mutant p53 proteins, referred to as gain of function (GOF) mutations [19, 20]. One notable GOF activity of mutant p53 is dysregulation and subversion of autophagy to promote chemoresistance and metastatic potential [21].

Disruptive p53 mutations have been further categorized into DNA contact (class I) and conformational (class II) mutations, depending on their effect on p53’s DNA binding domain (DBD). DNA contact mutants similar to those that affect Arg248 and Arg273 directly alter critical p53 residues involved in binding DNA without affecting DBD thermodynamic stability [22, 23]. By contrast, conformational mutants, such as those affecting Arg173, alter the conformation of p53’s DBD [23, 24]. In summary, p53 mutations come in many flavors and may induce changes to p53’s structure and function that are not recapitulated by other mutations.

Cancers may also disrupt p53 function by epigenetically downregulating the *TP53* locus [25] and enhancing p53 proteasomal turnover by amplifying *MDM2* [26, 27]. Due to p53’s importance in activating intrinsic apoptosis, p53 disruption in cancer abrogates efficacy of radiation and chemotherapy [28–30]. However, intrinsic apoptosis is only one of several activatable RCD pathways in cancer. Other RCD pathways, which we discuss in this review, may occur without p53. Due to p53’s paramount importance in maintaining homeostasis, p53 mutation promotes a dysregulated cellular environment that may enhance such “p53-independent” RCD pathways. To survive, cancers become addicted to the suppression of RCD pathways that would otherwise limit tumor progression. p53-independent RCD pathways and the mechanisms that cancers activate to suppress RCD represent valuable therapeutic targets in p53-mutant cancers. Treatment of p53-mutant cancers is especially relevant in cancer therapy because p53 mutation enhances the principal cause of cancer mortality, metastasis.

## p53 mutation and metastasis

p53 regulates several processes necessary for metastasis [28, 31]. Metastasis is a multistep process that results in the dissemination of a primary tumor to distal regions of the host organism. Metastasis occurs through sequential tumor invasion into neighboring tissue, intravasation into the vasculature, circulation, extravasation out of the vasculature, and, finally, colonization [32]. Metastasis is considered the predominant cause of cancer mortality, and identification of effective therapies for metastatic cancer is of utmost importance [33]. p53 limits activation of metastatic processes including epithelial–mesenchymal transition, disruption of the extracellular matrix, and enhancement of cell mobility.

p53 suppresses metastasis by regulating transcription factors that coordinate epithelial–mesenchymal transition (EMT). EMT refers to the dynamic, reversible process in which tightly bound epithelial cells undergo a phenotypic transformation into more migratory mesenchymal cells. EMT is characterized by the loss of adherens junctions and desmosomes, which are essential for epithelial cell adhesion. Cells that have undergone EMT exhibit enhanced cell mobility and invasion propensity [34]. EMT activation is a critical component of metastasis and is promoted by transcription factors such as Snail (*SNAI1*), Slug (*SNAI2*), Twist (*TWIST1*), and ZEB1/2. Wild-type p53 opposes EMT-promoting transcription factors in multiple ways. For example, p53-dependent transcription of *MDM2* enhances Snail and Slug proteasomal degradation, terminating EMT signaling [35, 36]. Additionally, p53-dependent expression of microRNAs, specifically miR34 and miR200 family members, represses Snail, Slug, ZEB1, and ZEB2 [37, 38]. p53 therefore suppresses metastasis by regulation of transcription factors that coordinate EMT.

p53 limits metastasis by promoting extracellular matrix (ECM) stability. Destruction of the ECM is a key metastatic process and facilitates tumor invasion, intravasation, and extravasation. ECM destruction is in part mediated by urokinase-type plasminogen activator (uPA) and tissue-type plasminogen activator (tPA), which are serine proteases that convert plasminogen to its active form, plasmin. Plasmin cleaves several proteins which form the ECM. p53 promotes ECM stability by repressing expression of uPA and tPA [39]. p53 also promotes expression of plasminogen activator inhibitor 1 (PAI-1), which inhibits uPA and tPA [39]. Matrix metalloproteases (MMPs) also contribute to ECM destruction, and p53 is thought to suppress ECM destruction by MMP regulation [31]. Moreover, p53 opposes cell migration in metastasis by activating transcription of caldesmon and phosphatase and tensin homolog (*PTEN*), which oppose podosome formation [40]. In contrast to wild-type p53, p53-mutant proteins promote metastasis by promoting ECM destruction and enhancing cell migratory structures [39, 41].

As discussed, p53 mutation potentiates metastasis by several mechanisms. p53 mutation is enriched in metastases relative to primary tumors across several cancer types [42]. Given the potentiation of metastasis and chemoresistance in cancers with mutant p53, it is crucially important to identify additional cell death pathways that may be pharmacologically activated. In the following review, we discuss four RCD pathways that may be activated to treat p53-mutant cancers, including E2F transcription factor 1 (E2F1)-dependent apoptosis, necroptosis, mitochondrial permeability transition driven necrosis, and ferroptosis. We review the mechanisms of RCD activation and execution, effects of p53 mutation on these RCD pathways, and current strategies for pharmaceutical activation of these RCD pathways in p53-mutant cancers.

## E2F1-dependent apoptosis with and without p53

While p53 mutation significantly limits apoptosis induction, cancers with mutant p53 may still undergo apoptosis downstream of E2F transcription factor 1 (E2F1). E2F1 is a member of the E2F family of transcription factors which serve as master regulators of the cell cycle [43]. E2F1 is hyperactivated in many cancers, promoting rapid proliferation. However, E2F1 activation also induces apoptosis as a fail-safe mechanism for limiting tumor formation when E2F1 is dysregulated. Supporting the role of E2F1 in apoptosis and tumor suppression, in vivo studies demonstrate that *E2F1* homozygous knockout mice spontaneously develop tumors, and thymocytes from these mice are resistant to apoptosis [44, 45]. Homozygous deletion of *E2F1* in p53 null mice results in greater tumor burden, demonstrating the functional significance of E2F1 in tumor suppression in the absence of p53 [46]. Unlocking the apoptotic potential of E2F1 could therefore provide an effective cancer-specific therapy, particularly in p53-mutant settings.

p53 mutation results in E2F1 hyperactivation by dysregulating control over E2F1 by pocket protein RB transcriptional corepressor 1 (RB1/pRB) and cyclin-dependent kinases (CDKs). Normally, pRB binds and inhibits E2F1. pRB phosphorylation by cyclin-dependent kinases (CDKs) releases E2F1 from pRB, resulting in transcription of E2F1 target genes [47]. Dysregulation of the CDK–pRB–E2F axis in cancers promotes cell cycle checkpoint escape and proliferation [48–50]. p53 mutation disrupts CDK and pRB signaling in cancer, resulting in constitutive activation of E2F1, leading to an enhanced DNA synthesis rate [51], replication stress [49], and G_1_/S transition [52].

Cell cycle control by E2Fs is coupled with ribosome biogenesis in the nucleolus. The nucleolus is a dynamic subnuclear condensate that is the site for ribosomal RNA (rRNA) transcription by RNA polymerase I (Pol I) [53]. Once processed, rRNA serves as the structural and catalytic core of the ribosome. In the nucleolus, E2F1 upregulates rRNA transcription by ribosomal DNA promoter binding [54]. E2F1 hyperactivation in cancer supports the increased demand for protein synthesis in rapidly dividing cells. Thus, E2F1 controls both cell cycle progression and ribosome biogenesis, making constitutive activation of E2F1 an attractive strategy for cancers to promote growth.

### E2F1-dependent apoptosis pathways

**Fig. 2:**
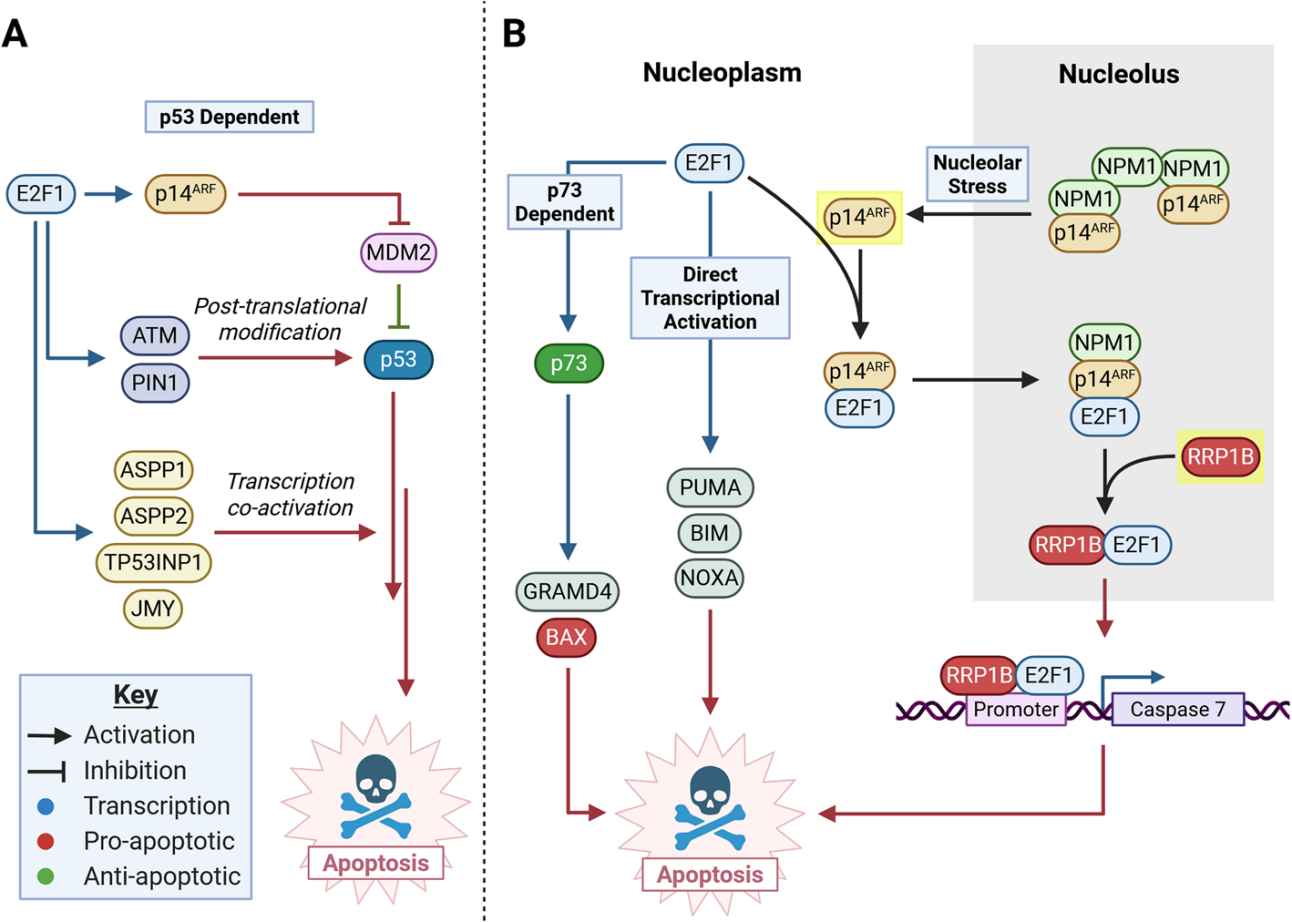
E2F1 activation of p53-dependent and independent apoptosis. Activation of E2F1 results in apoptosis by p53-dependent and p53-independent mechanisms. **A** In p53-dependent pathways, E2F1 enhances transcription of MDM2 inhibitor p14^ARF^, p53 posttranslational modifiers (*ATM*, *CHK2*, and *PIN1*), and transcription coactivators (*ASPP1*, *ASPP2*, *TP53INP1*, and *JMY*), which enhance p53-dependent apoptosis. **B** In p53-independent pathways, E2F1 enhances p73 transcription, a p53-homolog which transcribes *PUMA* and *BAX*, leading to MOMP and apoptosis. E2F1 directly transcribes proapoptotic genes *GRAMD4*, *BIM*, and *NOXA*, enhancing mitochondrial outer membrane permeabilization (MOMP) and inducing apoptosis. Nucleolar stress induced by DNA damage results in p14^ARF^ (highlighted) delocalization from the nucleolus to the nucleoplasm, resulting in recruitment and sequestration of nuclear E2F1 to nucleoli. Nucleolar E2F1 interacts with RRP1B (highlighted), resulting in enhanced transcription of caspase 7. Created in BioRender. Chung, J. (2025) https://BioRender.com/so661da

Apoptosis activation by E2F1 occurs in both p53-dependent and independent pathways (Fig. 2). In p53-dependent pathways, E2F1 activates transcription of p14^ARF^, which stabilizes p53 in response inhibiting MDM2 (Fig. 2A) [55, 56]. E2F1 positively regulates other activators of p53 such as ataxia telangiectasia mutated (ATM) and checkpoint kinase 2 (CHK2), which phosphorylate p53 and inhibit turnover by MDM2 [9, 57–59]. E2F1 also promotes p53-dependent apoptosis by transcriptional activation of p53 interaction partners *ASPP1*, *ASPP2*, *JMY*, *TP53INP1*, and *PIN1* [60–63]. Consistent with E2F1’s function upstream of p53, *E2F1* knockout abrogates p53-dependent apoptosis [64].

E2F1 may induce apoptosis in the absence of p53 by directly transcribing proapoptotic genes including *PUMA*, *BIM/BCL2L11*, and *NOXA* (Fig. 2B) [65]. Inhibition of E2F1-dependent transcription in p53^−/−^ mouse embryonic fibroblasts (MEFs) results in loss of PUMA expression and apoptosis in response to DNA damaging agents cisplatin and doxorubicin [65, 66]. Proapoptotic genes APAF-1 and caspases 3, 7, 8, and 9 are also directly transcribed by E2F1 [67–70]. Thus, E2F1 may induce mitochondrial apoptosis in p53-deficient settings by direct transcription of apoptosis effectors. E2F1 may also induce apoptosis by direct transcriptional activation of the p53-homolog p73 (Fig. 2B) [71]. p73 mediates chemosensitivity of tumors in the absence of p53 [72]. Notably, p73 is rarely mutated in cancers, suggesting that p73 activation could be a generally applicable cancer treatment strategy. p73 binds to p53 response elements, transactivating p53 targets including proapoptotic genes *PUMA* and *BAX* [73]. Furthermore, p73 transactivates genes that p53 does not such as death-inducing protein, DIP (*GRAMD4*) [74]. DIP promotes apoptosis by localizing to mitochondria and inhibiting the antiapoptotic BCL2 apoptosis regulator (BCL2) on the mitochondrial outer membrane [74]. Additionally, p73 performs transcription-independent functions in apoptosis [75]. p73 is activated by several chemotherapies, and silencing p73 results in chemoresistance [72]. Thus, p73’s ability to induce apoptosis might mediate chemosensitivity in the absence of p53.

Emerging evidence demonstrates that E2F1 may activate apoptosis downstream of nucleolar stress in a p53-independent manner (Fig. 2B). Nucleolar stress refers to a state where stresses including DNA damage and specific Pol I inhibition result in characteristic changes in nucleolar morphology and functional disruption of ribosome biogenesis [76]. When p53 is present, sufficient nucleolar stress results in p53-dependent apoptosis downstream of MDM2 inhibition by nucleolar proteins ribosomal protein L5 (RPL5), ribosomal protein L11 (RPL11), and p14^ARF^ (Fig. 1B) [77]. p14^ARF^, by shuttling between nucleoli and the nucleoplasm, performs a key role in activating apoptosis following nucleolar stress (Fig. 2B). p14^ARF^ normally resides in nucleolar assemblies with nucleophosmin, a mediator of nucleolar condensation [78, 79]. With nucleolar stress, which might occur owing to DNA damage or Pol I inhibition, p14^ARF^ transiently relocalizes to the nucleus (Fig. 2) [80]. p14^ARF^ nuclear localization results in sequestration of MDM2 to nucleoli, promoting p53 stabilization and activation [80, 81]. In addition to p14^ARF^, RPL5 and RPL11 also inhibit MDM2, activating p53-dependent apoptosis in response to nucleolar stress [82].

In the absence of p53, p14^ARF^ mediates apoptosis by activating E2F1. Similar to MDM2, E2F1 is recruited by p14^ARF^ to nucleoli in times of nucleolar stress [83, 84] (Fig. 2B). E2F1 nucleolar localization is promoted by ATM-dependent phosphorylation, which enhances the interaction between E2F1 and p14^ARF^ [84]. E2F1 nucleolar localization by p14^ARF^ might stabilize E2F1 by facilitating an interaction with MDM2 which stabilizes E2F1 [85]. Therefore, p14^ARF^ and nucleolar stress downstream of DNA damage control E2F1 localization and stability. E2F1 accumulation in the nucleolus by p14^ARF^ might enhance E2F1-dependent apoptosis by allowing E2F1 to interact with ribosomal RNA processing 1B (RRP1B) and other transcriptional coactivators (Fig. 2B). RRP1B is a direct transcriptional target of E2F1 and DNA damage leads to induction of both E2F1 and RRP1B [86]. RRP1B is a nucleolar protein that directly interacts with E2F1’s DNA-binding domain and enhances E2F1 transcription of proapoptotic genes *APAF-1*, caspase 3, and caspase 7 [86]. Interestingly, RRP1B also enhances E2F1-dependent rRNA promoter transcription [86]. Therefore, RRP1B interacts with E2F1 to potentiate both apoptosis and rRNA transcription in times of nucleolar stress.

Several other coregulators of E2F1 such as RRP1B have been described that may promote E2F1-dependent apoptosis in the absence of p53. These include Jab1 [87, 88], HCF-1/H3K4 methyltransferases [89], and TIP49 [90]. The nucleolar protein ribosomal protein L3 (RPL3) also regulates E2F1-dependent transcription in response to nucleolar stress [91]. Upon induction of nucleolar stress in p53^−/−^ cancer cells, RPL3 translocates to the nucleoplasm and indirectly represses E2F1-dependent transcription of cyclins [91]. Nucleolar stress induction in this setting also enhanced transcription of BAX, promoting apoptosis [91]. Taken together, accumulating evidence suggests that induction of nucleolar stress represses E2F1’s function in cell cycle progression while promoting E2F1-dependent apoptosis. Proapoptotic functions of E2F1 occur in p53^−/−^ cell lines and are promoted by E2F1 interactors such as RRP1B and RPL3. Nucleolar stress induction of E2F1-dependent apoptosis represents a highly promising strategy for treatment of p53-mutant cancers.

### Effects of p53 mutation on E2F1-dependent apoptosis

Effects of p53 mutation in both activation and suppression of E2F1-dependent apoptosis have been identified. As mentioned, p53 mutation leads to E2F1 hyperactivation by dysregulating pRB/CDK control over E2F1. Wild-type p53 normally activates transcription of p21 (*CDKN1A*), a CDK inhibitor that promotes pRB hypophosphorylation [92]. Hypophosphorylated pRB inhibits E2F1 transcription, resulting in p21-dependent cell cycle arrest downstream of p53 activation [92]. However, tumor-derived mutant p53 proteins are unable to transactivate p21, resulting in E2F1 hyperactivation [93]. Interestingly, several p53 mutants are able to transactivate p21 without activation of other p53-responsive genes that promote apoptosis [94]. Nonetheless, p53 mutation results in E2F1 hyperactivation in many cancers, and these cancers may be sensitive to E2F1-dependent apoptosis. However, p53 mutation simultaneously represses E2F1-dependent apoptosis to promote tumor survival.

p53 mutation results in repression of E2F1-dependent apoptosis by several pathways. Activation of wild-type p53 proteins promotes apoptosis by transcriptionally repressing forkhead box M1 (*FOXM1*). Several tumor-derived p53 mutants are unable to repress *FOXM1* transcription, leading to enhanced chemoresistance [95, 96]. Enhanced *FOXM1* expression in breast cancer cells promotes E2F1-dependent DNA damage repair and resistance to the DNA damage [96]. Similarly, p53 mutation results in enhanced transcription of sirtuin-1 (*SIRT1*), which abrogates E2F1-dependent apoptosis in p53-null lung cancer cells [97]. *SIRT1* enhancement in p53-mutant cells occurs owing to the inability of p53 mutant proteins to repress c-myc, a transcriptional activator of *SIRT1* [98]. Notably, SIRT1 and other sirtuin family members have been implicated in tumor suppression, supporting a context-dependent role of sirtuins that is not yet fully understood [99].

p53 mutation represses E2F1-dependent apoptosis by activation of the phosphoinositide-3-kinase (PI3K)/Akt survival pathway. The PI3K/Akt pathway is commonly hyperactivated in cancers, leading to tumor growth and apoptosis inhibition [100]. Activation of wild-type p53 represses the PI3K/Akt pathway, promoting cell death. p53-dependent repression of the PI3K/Akt pathway occurs in PTEN-dependent and independent manners [101, 102]. In contrast, tumor-derived p53 mutants activate the PI3K/Akt pathway, promoting cell survival and repressing E2F1-dependent apoptosis [103, 104]. One way that the PI3K/Akt pathway suppresses E2F1-dependent apoptosis is by activating DNA topoisomerase II binding protein 1 (TopBP1) [105, 106]. Activated Akt kinase phosphorylates TopBP1, allowing TopBP1 to oligomerize and interact with E2F1 [106]. TopBP1 oligomerization leads to recruitment of Brg1/Brm to E2F1-responsive promoters, downregulating E2F1 gene targets [105]. Furthermore, Jab1, which interacts with E2F1 and promotes apoptosis, is inhibited by PI3K activation [88]. Thus, PI3K/Akt pathway hyperactivation downstream of p53 mutation opposes activation of E2F1-dependent apoptosis.

p53-mutant proteins may also abrogate E2F1-dependent apoptosis by inhibiting p73 activation. While wild-type p53 does not interact with p73, the most common p53 point mutants R175H and R273H inhibit other p53 family members including p73 [107]. p53 R175H mutant proteins abrogate p73-dependent apoptosis activated by E2F1 [71]. In summary, p53 mutants may abrogate E2F1-dependent apoptosis by transcription independent mechanisms and transcriptional modulation of apoptosis regulators such as FOXM1, SIRT1, and the PI3K/Akt pathway.

### E2F1-dependent apoptosis in cancer therapy

Several small molecule inhibitors of FOXM1, SIRT1, and the PI3K/Akt pathway have been identified that promote chemosensitivity and apoptosis in p53-mutant cancers. FOXM1 inhibitors are currently being elucidated that promote chemosensitivity in a wide range of cancers [108, 109]. Interestingly, FOXM1 expression enhances resistance to other RCD pathways such as ferroptosis (discussed below in “Effects of p53 mutation on ferroptosis”), suggesting that FOXM1 inhibition could activate multiple RCD pathways in p53-mutant cancers. FOXM1 inhibitors are currently in preclinical development, and several synergistic combinations with chemotherapies have been demonstrated in vitro [110]. Several studies utilizing in vitro cell cultures and ex vivo cancer models also demonstrate that SIRT1 inhibitors may be effective treatments for inducing apoptosis and overcoming chemoresistance in cancer [111]. Despite promising preclinical evidence, clinical trials assessing the efficacy of SIRT1 inhibitors in cancer therapy are limited, possibly due to contradictory findings regarding SIRT1’s role as an oncogene or tumor suppressor. Nonetheless, SIRT1 inhibitors may be useful molecularly targeted cancer therapies with the further elucidation of SIRT1’s function. E2F1-dependent apoptosis may also be enhanced by inhibition of antiapoptotic pathways such as the PI3K/Akt pathway. Several PI3K pathway inhibitors have been approved for clinical use by the US Food and Drug Administration (FDA), and PI3K inhibitors are undergoing active development and testing. However, PI3K inhibitors suffer from severe adverse effects and variable clinical efficacy [112, 113]. Despite current limitations, inhibition of prosurvival pathways hyperactivated in mutant p53 cancers represent promising anticancer strategies.

E2F1-dependent apoptosis may also be induced for cancer therapy by activating E2F1 beyond the threshold required for apoptosis. High E2F1 activation is associated with transcription of apoptotic genes, while lower levels of E2F1 promote cell cycle progression [114]. E2F1 overexpression in glioma induces apoptosis and tumor suppression [115]. Furthermore, adenoviral gene transfer of E2F1 in melanoma xenografts on nude mice led to significant enhancement of tumor response to topoisomerase II inhibitors [116]. While these strategies promote apoptosis by enhancing E2F1 expression, other strategies have focused on preventing E2F1 degradation. For example, Shats et al. used a proteasome inhibitor to induce apoptosis in p53^−/−^ cell in vitro, an effect that was dependent on E2F1 [114]. Proteasome inhibitors such as bortezomib are approved for treatment of multiple myeloma and mantle-cell lymphoma [117]. Combining agents that enhance E2F1 expression with inhibitors of prosurvival pathways in p53-mutant cancers could yield highly potent therapies.

Given the role of E2F1 in responding to stress by interacting with RRP1B and other transcriptional coregulators, enhancement of proapoptotic E2F1 interactions might induce apoptosis. Drugs inducing nucleolar stress without damaging DNA, such as BMH-21 [118] and sempervirine [119], can activate both p53-dependent and independent apoptosis. In summary, E2F1-dependent apoptosis may occur through p53-independent pathways, and cancer therapies targeting E2F1 might be effective in treating p53-mutant cancers. Next, we discuss necroptosis, a cell death pathway that, unlike apoptosis, utilizes a caspase-independent pathway.

## Necroptosis in surveillance and treatment of p53-mutant cancers

**Fig. 3:**
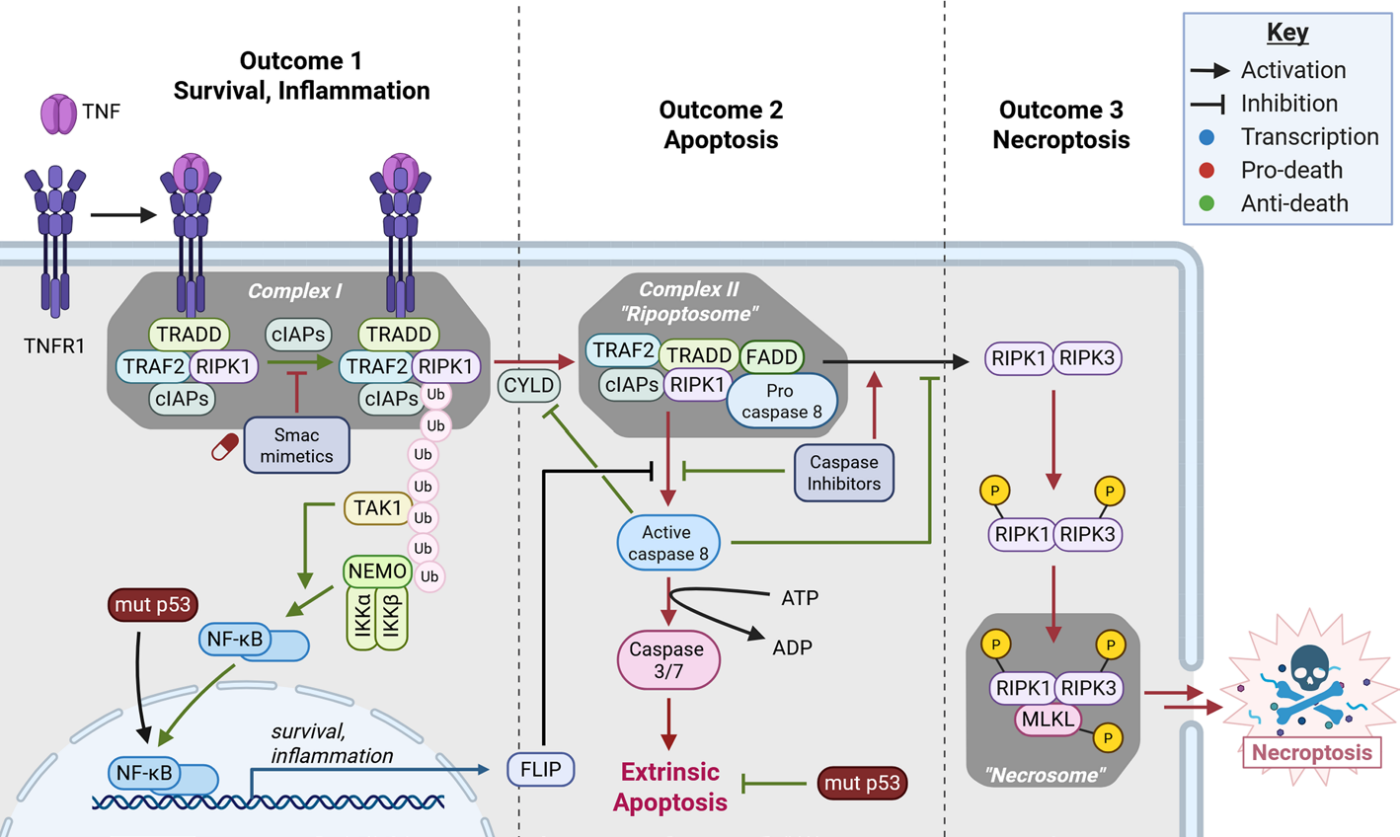
Tumor necrosis factor (TNF) receptor 1 induces inflammation, extrinsic apoptosis, and necroptosis. Ligation of tumor necrosis factor receptor 1 (TNFR1) results in recruitment of complex I, composed of TNFRSF1A associated via death domain (TRADD), tumor necrosis factor receptor associated factor 2 (TRAF2), receptor interacting serine/threonine kinase 1 (RIPK1), and cellular inhibitor of apoptosis proteins (cIAPs). cIAPs and linear ubiquitin chain assembly complex (LUBAC; not shown) ubiquitinate RIPK1, resulting in the activation of NF-κB and cell survival and inflammation. Cylid lysine 63 deubiquitinase (CYLD) deubiquitinates RIPK1, signaling conversion of the receptor-bound complex I to the cytosolic complex II. Complex II activates caspase-8, resulting in extrinsic apoptosis. Complex II activation and caspase-8-inhibition by FLICE-inhibitory protein (FLIP), genetic deletion, or caspase inhibitors promotes RIPK1 and RIPK3 heterodimerization and phosphorylation. RIPK1/RIPK3 heterodimers phosphorylate MLKL, resulting in necroptosis. Created in BioRender. Chung, J. (2025) https://BioRender.com/7g1own0

Necroptosis is a form of RCD associated with activation of receptor interacting serine/threonine kinase 3 (RIPK3) and culminating with formation of the necrosome (Fig. 3). Several stimuli have been shown to activate necroptosis, including toll-like receptor and interferon signaling [120]. Necroptosis is one potential outcome of death receptor (DR) binding to its cognate ligand. Activation of DRs by ligand binding leads to various outcomes including cell survival by nuclear factor kappa B (NF-κB) signaling, extrinsic apoptosis, and necroptosis (Fig. 3). Necroptosis has been implicated in surveillance of mutant p53 cells, and necroptosis can be induced pharmacologically to kill p53-mutant cancers.

### The necroptotic pathway

The core necroptotic machinery is largely independent of p53, and necroptosis may therefore be activated in tumor surveillance and treatment of p53-mutant cancers. The prototypical necroptotic pathway begins with activation of tumor necrosis factor receptor 1 (TNFR1). Tumor necrosis factor (TNF)-mediated activation of necroptosis begins with binding of TNF with TNFR1 (Fig. 3). Ligation of TNFR1 results in rapid formation of a lipid raft-bound signaling platform known as complex I, composed of TNFR1, TNFRSF1A associated via death domain (TRADD), tumor necrosis factor receptor associated factor 2 (TRAF2), and receptor interacting serine/threonine kinase 1 (RIPK1) [121–125]. Cellular inhibitor of apoptosis (cIAP) proteins, cIAP1, cIAP2, and X-linked inhibitor of apoptosis protein (XIAP), linear ubiquitin chain assembly complex (LUBAC), and a host of other ubiquitination regulators bind to RIPK1 and facilitate RIPK1 polyubiquitination [126–130]. RIPK1 polyubiquitination results in activation of TAK1 and the IKK signalosome, composed of NEMO, IKKα, and IKKβ, which activates NF-κB-dependent transcription [130–132]. NF-κB-dependent transcription promotes cell survival in part by transcription of *FLIP*, which inhibits caspase-8 [133–135]. Thus, complex I suppresses apoptosis by activating NF-κB transcription. Notably, p53 mutation results in sustained NF-κB activation, which might promote caspase-8 inactivation and necroptosis induction (further discussed below in “Effects of p53 mutation on necroptosis”).

Conversion of complex I to complex II signals a switch in function from cell survival to an RCD pathway known as the *extrinsic pathway of apoptosis* (Fig. 3). Though the factors that control the switch between complex I and II are incompletely understood, complex II formation is influenced by RIPK1 ubiquitination. As mentioned, cIAPs and LUBAC promote RIPK1 ubiquitination and subsequent NF-κB activation. In contrast, cylid lysine 63 deubiquitinase (CYLD) deubiquitinates RIPK1 and promotes complex II formation [136–138]. Complex II is formed following TRADD/TRAF2/RIPK1 dissociation from membrane-bound TNFR1 into the cytosol [133]. TRADD dissociation from TNFR1 exposes the TRADD death domain, resulting in the recruitment of Fas associated via death domain (FADD) and caspase-8 [133]. This cytosolic complex including FADD and caspase-8 is referred to as complex II or the *ripoptosome*. Complex II activates caspase-8, resulting in caspase-dependent extrinsic apoptosis [136, 139].

RIPK1 may induce necroptosis when caspase-8 is inactivated. Caspase-8 normally suppresses necroptosis by cleaving RIPK1 [140]. However, when caspase-8 is inactivated, RIPK1 undergoes autophosphorylation, leading to the recruitment and activation of RIPK3 [141–143]. Such caspase-8 inactivation may occur either by genetic deletion or pharmacological inhibition with caspase inhibitors [144]. Caspase-8 autocleavage has also been implicated in necroptosis induction [145]. Activated RIPK1/RIPK3 heterodimers recruit and phosphorylate mixed lineage kinase domain-like pseudokinase (MLKL), leading to necroptosis by an undetermined mechanism [143, 146, 147]. Current evidence suggests that phosphorylated MLKL forms amyloid-like polymers that lead to plasma membrane permeabilization [148–150]. Alternatively, MLKL polymers might mediate lysosomal permeabilization, resulting in leakage of proteases and subsequent membrane permeabilization [150]. In summary, the engagement of RIPK1 in complex I, complex II (the ripoptosome), and the necrosome mediates the different cell fates following TNFR1 receptor binding. Complex I mediates NF-κB-dependent survival and inflammation. Complex II activation results in apoptosis. When caspase-8 is inactivated, formation of the necrosome results in necroptosis. Regulatory factors, such as cIAPs and CYLD, mediate the interconversion of these complexes and control their respective functions. p53 mutation, discussed below, results in the activation of necroptosis as a tumor surveillance mechanism and the modulation of necroptosis downstream of NF-κB activation.

### Effects of p53 mutation on necroptosis

p53 mutation promotes the induction of necroptosis in response to extracellular cues as a mode of early tumor surveillance and elimination. p53 R175H and R273H mutant cells undergo necroptosis and basal extrusion when surrounded by normal epithelia [151]. Necroptosis of p53-mutant cells is inhibited by necrostatin-1, a necroptosis inhibitor, and RIPK3 silencing [151]. Furthermore, p53-mutant cells survive when surrounded with transformed cells, suggesting that tissue transformation is a prerequisite for the survival of p53-mutant cells during cancer development [151]. While mechanistic understanding of the extracellular factors that induce necroptosis in p53-mutant cells remains elusive, the authors speculate that the induction of necroptosis in p53-mutant cells surrounded by normal epithelium could explain why p53 mutations are more common in mid-to-late stage compared with early stage in most solid tumors [151].

One extracellular cue that induces necroptosis in p53-mutant cells is progesterone. Progesterone induces necroptosis of p53-deficient cells in the fimbrial epithelium of the fallopian tube [152]. RIPK1 expression is high in p53^−/−^ but not p53^+/+^ oviduct fimbriae during diestrus, and progesterone treatment enhances RIPK1 accumulation and necroptosis in these p53^−/−^ cells [152]. Necroptosis may therefore prevent high-grade serous ovarian cancer by eliminating p53-mutant cells prior to cancer development. p53 mutation also promotes necroptosis in response to sirtuin 3 (SIRT3) activation. Overexpression of SIRT3 in p53-mutant lung cancer cells leads to proteasomal degradation of mutant p53 and induction of necroptosis [153]. Overexpression of SIRT3 leads to downregulation of caspase-8 and upregulation of RIPK3, MLKL, and phospho-MLKL [153]. Furthermore, expression of SIRT3 impedes growth of p53-mutant mouse xenografts [153]. Emerging evidence suggests that necroptosis induction by hormone signaling and sirtuin 3 are related, though the relationship remains unclear [154–156]. In summary, necroptosis may occur in p53-mutant and deficient settings downstream of progesterone signaling and sirtuin 3 activation. Further study is required to assess whether these pathways may be pharmacologically activated for cancer therapy.

While p53 mutation seems to sensitize cancer cells to necroptosis induction in response to extracellular cues, the mechanisms underlying these observations remain poorly understood. One explanation for why p53-mutant cancers undergo necroptosis could be related to dysregulation of the NF-κB pathway, which is augmented and chronically activated in p53-mutant cancers [157, 158]. Such NF-κB activation has been demonstrated to enhance inflammation and promote dedifferentiation and metastasis [159]. NF-κB activation is canonically associated with a prosurvival response that suppresses apoptosis and necroptosis [160]. However, the relationship between NF-κB activation and necroptosis induction remains poorly understood, particularly in the context of p53 mutation, and NF-κB activation downstream of p53 mutation might promote necroptosis in certain settings. As mentioned, NF-κB transcriptionally activates c-FLIP, which inhibits caspase-8 activation [133–135]. NF-κB-dependent transcription of c-FLIP might promote necroptosis by inactivating caspase-8, which would otherwise cleave the critical necroptosis mediators RIPK1 and RIPK3 [140, 161]. Chronic NF-κB activation by mutant p53 could explain why p53-mutant cells are sensitized to necroptosis in response to extracellular cues. Interestingly, activation of necroptosis has been implicated in the termination of cytokine production, therefore blunting inflammation [162]. Rapid induction of necroptosis blunts inflammation by terminating cytokine-producing cells in vitro and in vivo [162]. Thus, tipping the balance of NF- κB away from inflammation and toward necroptosis in cancer therapy may limit inflammation and TME remodeling. While there is still much to learn about the effect of p53 mutation on necroptosis induction, the induction of necroptosis could be a tenable strategy for the treatment of p53-mutant cancers. Several necroptosis inducers are now emerging as possible anticancer therapies.

### Necroptosis in cancer therapy

Because cancers with mutation of p53 can efficiently undergo necroptosis, activation of necroptosis may be useful in settings where apoptotic capacity is diminished by p53 mutation. Several agents have been identified that induce necroptosis in cancers [163]. Conventional chemotherapies such as doxorubicin and etoposide activate both apoptosis and necroptosis in cultured cancer cells, and the efficacy of these agents is reduced with silencing *RIPK3* and *MLKL* [164]. However, targeted therapies for the induction of necroptosis are now coming of age.

Diablo IAP-binding mitochondrial protein (DIABLO or SMAC) mimetics such as birinapant antagonize cIAP proteins, promoting complex II-dependent extrinsic apoptosis and necroptosis [165–168] (Fig. 3). Birinapant has been studied in several human clinical trials, and birinapant is generally well-tolerated, bioavailable, and on-target [169]. Birinapant induces necroptosis in head and neck squamous cell carcinomas (HNSCC), which are more frequently mutated in p53 than other tumor types [170, 171]. HNSCC necroptosis by birinapant is dependent on RIPK3 expression and enhanced with caspase-8 silencing [171]. Despite strong preclinical evidence, response to birinapant in human clinical trials is limited (nos. NCT02587962, NCT01681368, and NCT01188499) [169, 172, 173]. The limited efficacy of birinapant could be a result of applicability limited to cancers with a functional necroptotic pathway and birinapant’s general inefficacy as a single agent. Lalaoui et al. found that birinapant efficacy was dependent on sufficient TNF abundance and a competent death pathway [167]. Furthermore, estrogen receptor-positive tumors were resistant to birinapant [167].

DIABLO mimetic resistance could be mediated by the epigenetic regulation of necroptosis inducers such as RIPK3. *RIPK3* methylation status differs between primary and recurrent tumors, demonstrating that the necroptotic pathway is dynamically modulated by epigenetic modification throughout the cancer course [174]. Therefore, biomarker-driven design may be necessary to enhance DIABLO mimetic efficacy. One clinical trial utilized biomarker-driven patient selection when applying an IAP antagonist, LCL161, with paclitaxel (no. NCT01617668) [175]. Triple-negative breast cancers with an increased TNFα gene signature were more likely to respond to combination treatment with LCL161 and paclitaxel compared with paclitaxel monotherapy [175]. In addition to DIABLO mimetics, other compounds have been identified that induce necroptosis in p53-mutant cancers. Shikonin, a naturally occurring pigment from *Lithospermum erythrorhizon*, induces necroptosis in osteosarcoma and non-small cell lung cancer cell lines [176, 177]. Rigorous studies demonstrating the efficacy and specific mechanism of shikonin-induced necroptosis remain limited. Nonetheless, the application of shikonin and its derivatives may represent a feasible treatment for p53-mutant cancers upon further study.

In summary, necroptosis represents an RCD pathway that occurs downstream of death receptor signaling and in p53-mutant and deficient settings. Necroptosis functions as a tumor surveillance mechanism; however, necroptosis mediators have diverse functions and are subject to dynamic epigenetic regulation that could facilitate therapeutic resistance. Clinical trials applying necroptosis inducers, such as DIABLO mimetics, have demonstrated good tolerability but poor efficacy, highlighting the need for precise biomarker-driven application of necroptosis inducing agents. Next, we discuss mitochondrial permeability transition-driven necrosis, an RCD pathway with significant crosstalk with necroptosis.

## MPT-driven necrosis in p53-mutant cancers

Similar to necroptosis, mitochondrial permeability transition (MPT)-driven necrosis results in membrane permeabilization and a characteristic necrotic morphotype with hallmarks including cellular and organellar swelling [178]. Emerging evidence demonstrates that MPT-driven necrosis and necroptosis are interrelated [179]. However, MPT-driven necrosis is distinct from necroptosis. In necroptosis, membrane permeabilization occurs downstream of MLKL phosphorylation, whereas MPT-driven necrosis is associated with severe bioenergetic stress and eventual inability for the cell to cope [180]. Both RCD pathways may be activated in p53-mutant cancers for treatment purposes. Here, we discuss the mechanism of MPT-driven necrosis and therapeutic induction of MPT-driven necrosis in p53-mutant cancers.

### Induction of MPT-driven necrosis

**Fig. 4:**
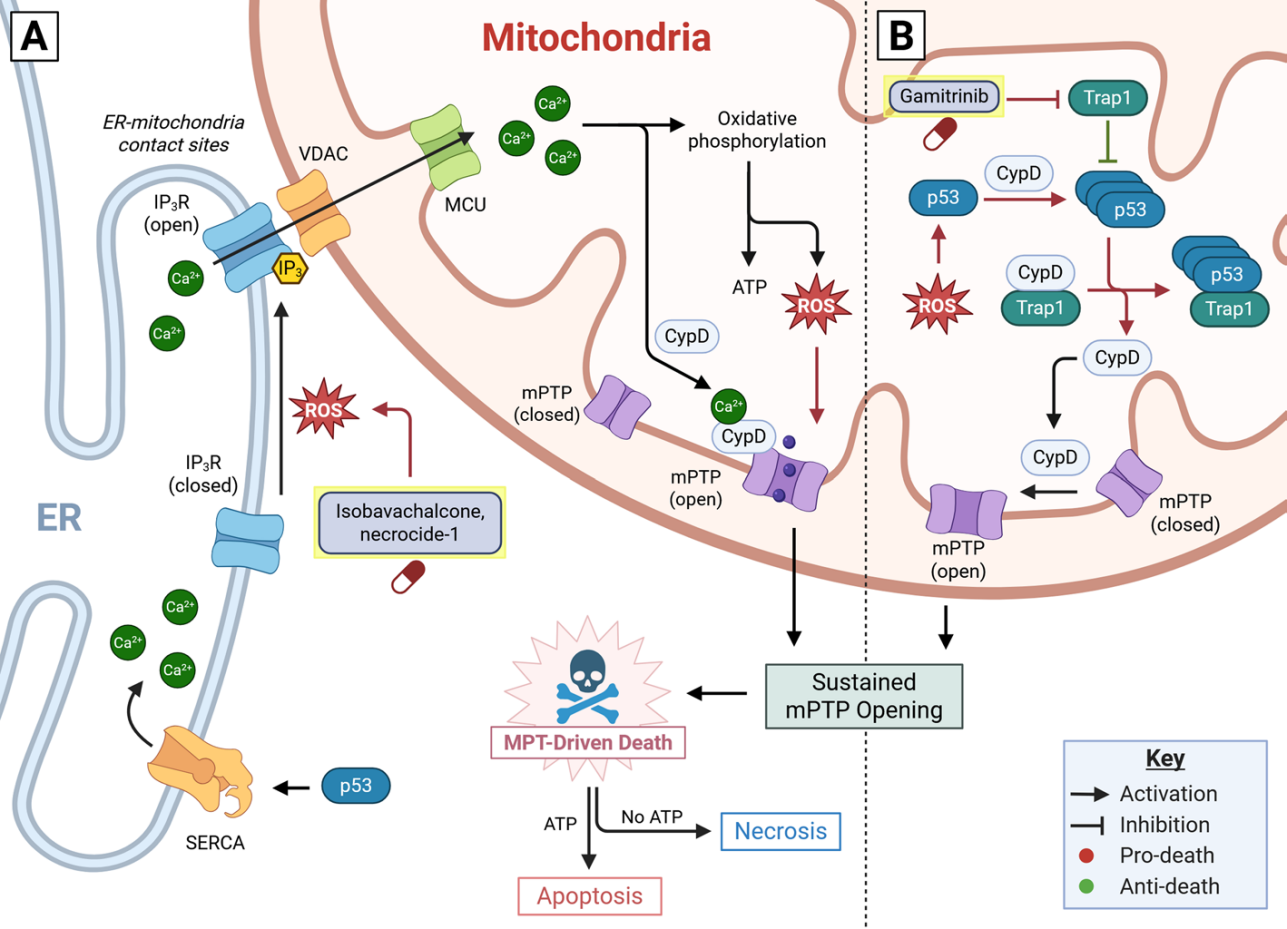
Mitochondrial permeability transition-driven necrosis and apoptosis. Mitochondrial permeability transition (MPT)-driven death occurs through **A** calcium- and **B** p53-dependent pathways. **A** In the calcium-dependent pathway, exposure of mitochondria to calcium results in excessive mitochondrial permeability transition pore (mPTP) opening, leading to cell death. Organelles including the endoplasmic reticulum (ER) serve as calcium stores and regulate intracellular calcium. p53 enhances ER calcium abundance by activating sarc/ER calcium ATPase (SERCA) pumps. Calcium is released from the ER by opening of inositol 1,4,5-triphosphate receptors (IP_3_R), which occurs in response to ligand (IP_3_) binding and elevated reactive oxygen species (ROS). Influx of calcium to the mitochondria matrix, which is mediated by calcium import channels (VDAC and MCU) at ER-mitochondrial contact sites, accelerates oxidative phosphorylation, enhancing ATP synthesis and ROS production. Increased mitochondrial ROS and calcium promote cyclophilin D (CypD)-dependent mPTP opening. MPT-dependent death resembles a necrotic morphotype if ATP is unavailable or an apoptotic morphotype if both mitochondrial outer membrane permeabilization (MOMP) and sufficient ATP are present with MPT. Isobavachalcone and necrocide-1 are two drugs that promote ROS and activate MPT-driven death in p53-mutant cancers. **B** MPT may also occur in a p53-dependent pathway, wherein p53 localizes to mitochondria following stabilization (by stresses including ROS) and aggregates. p53 aggregation is promoted by CypD, and p53 aggregates bind to heat-shock protein (HSP) complexes, liberating CypD. Liberated CypD can promote further mitochondrial p53 aggregation and MPT, leading to cell death. Gamitrinib promotes p53-dependent MPT-driven death by inhibiting TRAP1, which otherwise opposes p53 aggregation. Created in BioRender. Chung, J. (2025) https://BioRender.com/3huyrjs

While MPT-driven necrosis may occur independently of p53, p53 modulates MPT triggers including calcium and ROS (Fig. 4A). MPT-driven necrosis refers to necrotic death that occurs due to irreversible opening of the mitochondrial permeability transition pore (mPTP). The mPTP is a putative Ca^2+^-gated channel that spans the mitochondrial inner membrane (IMM) [181–183]. Mitochondrial permeability transition (MPT) refers to mitochondrial IMM permeabilization due to the opening of the mPTP. MPT was first demonstrated in the 1970 s by Hunter and Haworth, who demonstrated that mitochondria undergo substantial permeabilization and swelling when exposed to calcium [181, 184]. Calcium-permeabilized mitochondria facilitate the nonspecific transfer of solutes up to 1500 Daltons across the IMM, leading to membrane depolarization [181]. With irreversible mPTP opening and complete loss of mitochondrial membrane polarity, F_1_F_0_ ATP (F-ATP) synthase may hydrolyze ATP rather than synthesizing it [185]. Ultimately, sustained mPTP opening leads to a severe and irreparable ATP crisis, impaired maintenance of ion balance by ATP-dependent ion transporters, and necrosis soon after [178, 186]. mPTP opening is also associated with induction of apoptosis. Mitochondrial rupture can occur following MPT, resulting in cytochrome *c* release and apoptosis [187].

The molecular components of the mPTP and the mechanism of mPTP opening remain unclear despite intense research efforts [183]. F-ATP synthase [188], adenine nucleotide translocator (ANT) [189], and voltage-dependent anion channels (VDAC) [190] have been suggested to make up the mPTP. Current efforts are focused on F-ATP synthase and ANT as comprising the mPTP on the mitochondrial inner membrane, with VDAC and BCL2 family members facilitating outer mitochondrial membrane permeability and having a regulatory role on mPTP opening [183]. Despite controversy surrounding the mPTP, researchers agree that cyclophilin D (CypD) regulates mPTP opening. CypD is a peptidyl prolyl isomerase that interacts with ANT [191, 192] and F-ATP synthase [193] and facilitates mPTP opening in response to Ca^2+^ stimulation [194]. Loss of CypD abrogates Ca^2+^-dependent MPT [195]. Researchers commonly apply cyclosporine A (CsA), a CypD inhibitor, and bongkrekic acid, an ANT inhibitor, to explore functional relationships with the mPTP and MPT [196–198].

Activation of CypD and the MPT process are controlled by intracellular calcium and ROS levels, which in turn are modulated by p53 (Fig. 4A). Calcium homeostasis is maintained in lysosomes, endoplasmic reticulum (ER), and mitochondria. These organelles serve as intracellular calcium stores. Activation of inositol 1,4,5-triphosphate receptors (IP_3_R) and ryanodine receptors on the ER surface results in Ca^2+^ efflux from the ER into the cytosol or directly onto mitochondria Ca^2+^ influx channels near ER-mitochondria membrane contact sites (ERMCS) [199]. A proportion of intracellular p53 has been demonstrated to localize to ERMCS, promoting Ca^2+^ influx by ATPase sarcoplasmic/endoplasmic reticulum Ca^2+^ transporting 2 (SERCA2/ATP2A2) pumps [200]. Furthermore, p53 is a known regulator of intracellular ROS. ROS sensitizes Ca^2+^-release from the ER by oxidizing IP_3_Rs [201]. Cytosolic ROS accumulation therefore results in Ca^2+^ release from the ER and exposure of mitochondria to Ca^2+^. Influx of Ca^2+^ into the mitochondrial matrix, dependent on VDAC and MCU mitochondrial calcium import channels, activates oxidative phosphorylation and mitochondrial ROS production [202, 203]. Increased mitochondrial ROS results in the oxidation of solvent-exposed thiol groups on CypD and ANT, activating MPT [204, 205] (Fig. 4A). In summary, mPTP opening is stimulated by ROS and calcium, which are mutually dependent [206]. mPTP opening relieves Ca^2+^ accumulation in mitochondria and uncouples oxidative phosphorylation, decreasing mitochondrial ROS [207, 208]. Thus, MPT is thought to serve a protective role in limiting ROS-induced damage to mitochondria. However, sustained mPTP opening leads to the irreparable loss of mitochondrial function and cell death. p53 mutation leads to substantial changes to MPT regulation and may therefore represent a therapeutic vulnerability in p53-mutant cancers.

### Effects of p53 mutation on MPT-driven necrosis

Tumor-derived p53 mutants enhance cellular ROS by several mechanisms, including the enhancement of ROS production [209]. Mutant p53 proteins enhance mitochondrial superoxide production by a sestrin-1 dependent mechanism [210]. Mutant p53 proteins also enhance NADPH oxidase 4 (NOX4) expression, leading to increased ROS production. In contrast, wild-type p53 suppresses NOX4 [211]. Mutant p53 proteins also enhance cellular ROS by inhibiting ROS scavenging. For example, mutant p53 R273H attenuates the activation of the NF-E2-related factor 2 (NRF2) transcription factor, a major regulator of the antioxidant response [212, 213]. While ROS enhancement and tolerance in p53-mutant cancers is thought to promote tumorigenesis, impaired ROS regulation due to p53 mutation is now recognized as an attractive therapeutic target in p53-mutant cancers. Increased ROS promotes MPT, sensitizing p53-mutant cancers to MPT-driven cell death.

p53 mutation also results in suppression of MPT-driven cell death. Mutation of p53 impairs calcium signaling at ER-mitochondria contact sites (ERMCS). As discussed, ERMCS function in ROS-dependent MPT through calcium efflux from ER to mitochondria. Giorgi et al. showed that wild-type p53 accumulates at ERMCS and promotes mitochondrial permeabilization in response to ROS and DNA damage induction [200]. In contrast, tumor-derived p53 mutants were unable to recapitulate p53-dependent ERMCS calcium signaling, rendering p53-mutant colon cancers cells resistant to ROS induction [200]. Mechanistically, wild-type p53, but not mutant p53, was observed to enhance SERCA pump activity, promoting ER Ca^2+^ abundance and mitochondrial permeabilization in response to ROS [200]. p53 mutants are also unable to promote ER-mitochondrial calcium signaling by transcriptional activation of the tumor suppressor gene *PTEN*. Normally, PTEN promotes ER-mitochondrial calcium signaling by modulating IP_3_R activity [214]. Wild-type p53 transactivates *PTEN*, promoting ER-mitochondrial calcium flux [102]. However, mutant p53 cells are unable to transactivate *PTEN*, impairing ER-mitochondrial calcium signaling [102].

p53 mutation may also influence a transcription-independent function of p53 in mPTP opening (Fig. 4B). Vaseva et al. demonstrated that mPTP opening in response to ROS insult can occur owing to a direct interaction between p53 and CypD [215]. Upon accumulation of p53 by ROS, p53 mitochondrial aggregates enhance CypD activation of mPTP opening [215]. p53 knockout and cyclosporine A (CsA) treatment both ablate MPT-driven necrosis following oxidative challenge in MEFs and colorectal cancer cells [215]. Further investigation showed that induction of MPT-driven necrosis could also be stimulated by gamitrinib, an inhibitor of the mitochondrial chaperone TNF receptor associated protein 1 (TRAP1/HSP75) [216]. Induction of MPT by gamitrinib was dependent on presence of p53 and CypD [216]. These studies suggest that mPTP opening is dependent on p53 translocation and aggregation in mitochondria, which is enhanced by CypD and TRAP1 inhibition (Fig. 4B). While these studies demonstrate a transcription-independent function of p53 in MPT, the effect of p53 mutation in this pathway is unclear. Tumor-derived mutant p53 proteins were associated with higher binding to CypD in vitro compared with wild-type p53 [216]. These observations could be due to increased exposure of p53’s DNA binding domain (DBD) to CypD by point mutations that reduce p53 structural stability, such as R175H [216]. Consistent with this hypothesis, the p53 DBD was identified as the region mediating interactions with CypD. If mutant p53 proteins are competent in mPTP opening, p53-mutant cancers with high basal ROS levels could be especially vulnerable to MPT induction due to the enhanced interaction of mutant p53 proteins with CypD. Indeed, several p53 missense mutants are prone to aggregation [217]. However, the p53 domain mediating its interaction with CypD is controversial, and Zhao et al. demonstrated that p53’s intrinsically-disordered N-terminal domain binds to CypD with much greater affinity than the DBD [218].

### MPT-driven necrosis in cancer therapy

Small molecules are now emerging that induce MPT-driven death for cancer therapy. As mentioned above, gamitrinib, a TRAP1 inhibitor, was shown to induce MPT-driven necrosis in a p53-dependent manner (Fig. 4B) [216]. Despite the apparent role of p53 in facilitating mPTP, experimental therapeutics can induce MPT-driven necrosis in cancers without p53. For example, isobavachalcone and its derivatives induce MPT-driven necrosis in both p53 wild-type and null cancer cells with similar efficacy [219, 220]. Another compound, necrocide-1, effectively kills both p53-mutant and wild-type breast cancer cells by stimulating MPT-driven necrosis [221]. Necrocide-1 efficacy is associated with high basal ROS level, supporting the premise that high basal ROS might sensitize cancers to MPT-driven necrosis [221]. In acute lymphoblastic leukemia, enhancement of MPT by resveratrol, an F-ATP synthase inhibitor, and CsA, an inhibitor of CypD, were shown to enhance cell death in both p53-mutant and wild-type cancer cells [222]. In this study, CsA was associated with increased mitochondrial depolarization with resveratrol treatment, whereas other studies demonstrate that CsA limits membrane depolarization by inhibiting CypD. Promotion of membrane depolarization with CsA treatment might be due to CypD-independent effects of CypD [223]. PK11195, a promoter of mPTP opening, also increased membrane depolarization with resveratrol [222]. These findings further support MPT induction in suppressing p53-mutant cancers.

Human clinical trials assessing isobavachalcone and necrocide-1 anticancer properties are not yet available. Resveratrol, however, is under active clinical investigation for several indications including cancer, cardiovascular disease, and obesity [224]. Micronized resveratrol is bioavailable and well-tolerated, with only minor adverse effects. However, human clinical trials have found that resveratrol is largely inefficacious at limiting tumor progression (nos. NCT00920803 and NCT00920556) [225, 226]. One possible explanation for the limited efficacy of resveratrol in cancer therapy is that resveratrol activates SIRT1 [227]. SIRT1 (discussed in “E2F1-dependent apoptosis in cancer therapy”) has been demonstrated to promote cancer chemoresistance. While preclinical studies of resveratrol, isobavachalcone, and necrocide-1 demonstrate their promise in MPT induction, further study is required to confidently apply these agents as cancer therapies.

In summary, mitochondrial permeability transition (MPT), which occurs with the opening of the mitochondrial permeability transition pore (mPTP), plays an important role in induction of cell death. Sustained mPTP opening results in either apoptosis or necrosis, depending on outer mitochondrial membrane permeabilization and ATP abundance. p53 mutation modulates mPTP opening indirectly by regulating intracellular ROS and suppressing ER-mitochondrial calcium signaling. p53 can also induce MPT through a transcription-independent function with TRAP1 in mitochondria. Several MPT inducers effectively kill p53-mutant cancer cells; however, further investigation of tolerability and efficacy in treating p53-mutant cancers is necessary. Next, we discuss ferroptosis, which, similar to MPT-driven necrosis, is substantially driven by ROS and p53.

## Ferroptosis modulation by p53 mutation

**Fig. 5:**
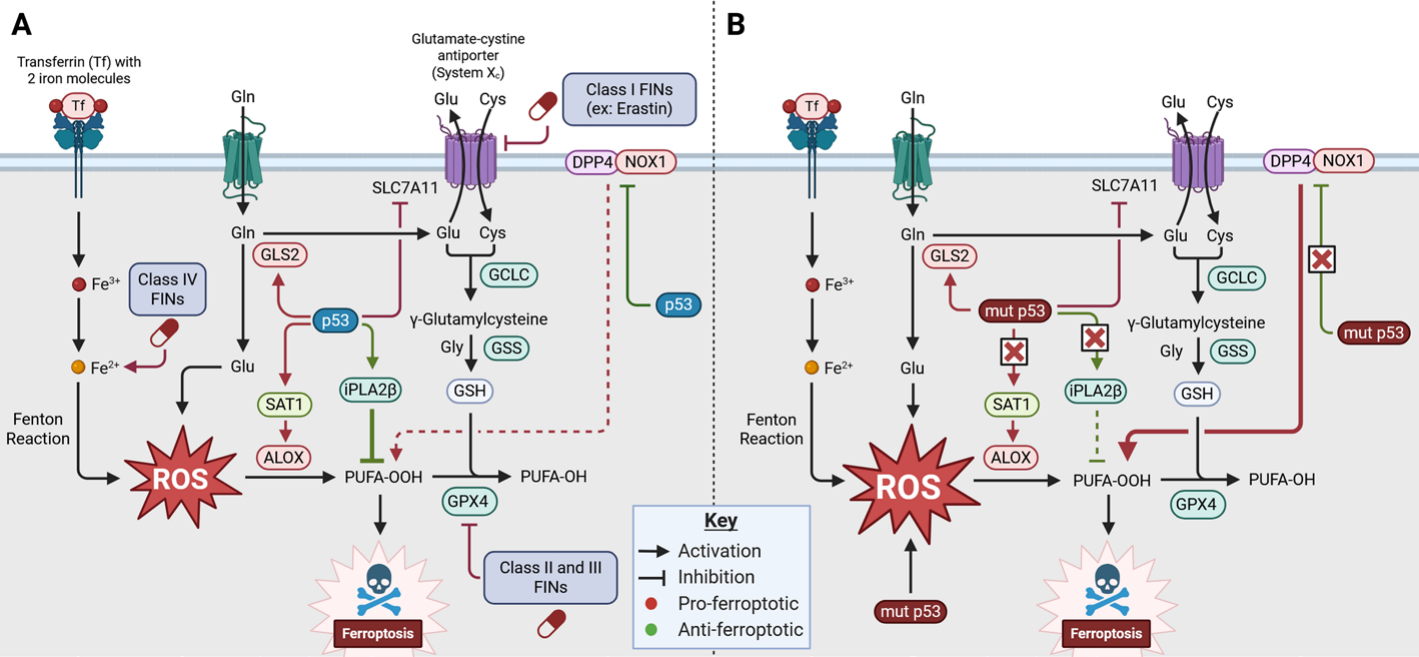
Ferroptosis activation by p53 and ferroptosis inducers. Ferroptotic pathways in **A** wild-type and **B** mutant p53 settings. In both settings, intracellular iron and ROS contribute to lipid peroxidation by enhancing the Fenton reaction and arachidonate lipoxygenase (ALOX) oxidation of polyunsaturated fatty acids (PUFAs). Excessive lipid peroxidation leads to ferroptotic cell death. Lipid peroxides are detoxified by glutathione peroxidase 4 (GPX4), which consumes glutathione (GSH). **A** Activation of wild-type p53 promotes ferroptosis by transcriptional activation of glutaminase 2 (GLS2) and spermidine/spermine N1-acetyltransferase 1 (SAT1), which promote ROS production and lipid peroxidation, respectively. p53 represses *SLC7A11* transcription, a component of system xc^-^, promoting ferroptosis by limiting glutathione (GSH) production. Activation of wild-type p53 may also oppose ferroptosis by transactivation of phospholipase A2β (iPLA2β) and limitation of membrane-bound dipeptidyl peptidase 4 (DPP4). Ferroptosis inducers (FINs) are drugs that enhance ferroptosis by acting on various steps of the ferroptotic pathway. Class I FINs inhibit system xc^−^. Class II and III FINs inhibit GPX4. Class IV FINs enhance oxidizing iron and lipid peroxidation. **B** In contrast to wild-type p53, mutant p53 proteins promote ferroptosis by increasing basal ROS. Mutant p53 proteins lose the ability to transactivate iPLA2β and limit membrane-bound DPP4, resulting in increased lipid peroxidation and ferroptosis. In contrast, mutant p53 proteins may limit ferroptosis compared with wild-type p53 owing to the inability of mutant p53 to transactivate *SAT1*. Both wild-type and mutant p53 perform functions that oppose and promote ferroptosis induction, highlighting the importance of the cellular context in ferroptosis activation. Created in BioRender. Chung, J. (2025) https://BioRender.com/te3smck

Ferroptosis is a recently discovered cell death pathway that has received a flurry of attention for its therapeutic potential in combating p53-mutant cancers. Ferroptosis is an iron-dependent, nonapoptotic form of cell death that is mediated by overwhelming ROS-dependent peroxidation of polyunsaturated fatty acids (PUFAs) (Fig. 5) [228, 229]. Ferroptosis is distinguished morphologically from other forms of cell death in that cells undergoing ferroptosis lack the hallmarks of other cell death modalities such as condensation of chromatin (apoptosis), organelle swelling (necrosis), and double-membraned vesicles (autophagy) [228]. Rather, ferroptotic cells appear shrunken prior to plasma membrane rupture [228]. Inhibitors of other RCD pathways are ineffective at preventing ferroptosis, and ferroptotic cells do not display ATP depletion as is characteristic of MPT-driven necrosis [228].

### Ferroptosis mechanism

While PUFA peroxides are normal byproducts of metabolism found in all cells, the enhanced production of PUFA peroxides or the inability for the cell to detoxify PUFA peroxides results in ferroptotic cell death. Lipid peroxides form when lipid alcohols interact with free hydroxyl radicals produced by cellular respiration, enzymes including P450 oxidoreductases, and Fenton reaction with intracellular iron. Excessive PUFA peroxidation is normally suppressed by antioxidant enzymes such as glutathione peroxidase 4 (GPX4), which requires glutathione (GSH) as a cofactor [230, 231]. Glutathione synthesis requires cystine imported by system xc^−^, an amino acid antiporter that shuttles cystine into the cell (Fig. 5) [231, 232]. Dysfunction of either GPX4 or system xc^−^ results in ferroptosis due to enhanced PUFA peroxidation. GPX4 and system xc^−^ are regulated by p53 and ferroptosis can occur downstream of p53 activation (Fig. 5A). Jiang et al. demonstrated that activation of p53 in MEFs results in the transcriptional repression of solute carrier family 7 member 11 (SLC7A11), a key component of system xc^−^ [233]. Similarly, wild-type p53 expression in breast cancer and osteosarcoma cells was associated with decreased SLC7A11 [234]. p53-dependent repression of system xc^−^ results in impaired GSH production, leading to accumulation of toxic fatty aldehydes and ferroptosis [229]. Indeed, silencing p53 in breast cancer and osteosarcoma cells promotes ferroptosis resistance [234]. Ultimately, p53-dependent inhibition of system xc^−^ represents a potent tumor-suppressive pathway. For example, many cancers upregulate SLC7A11 to support GSH production, thereby limiting the toxic accumulation of ROS [235]. Additionally, p53 transactivates GLS2, a glutaminase that promotes ferroptosis induction [236, 237]. While the p53/SLC7A11 axis was the first ferroptosis pathway demonstrated, other p53-dependent ferroptosis pathways have been described since [238].

Ferroptosis can be activated with small-molecule inducers of ferroptosis (FINs). Class I FINs such as erastin inhibit system xc^−^, while FINs belonging to other classes target GPX4 or iron regulation (Fig. 5) [228]. While GPX4-dependent PUFA detoxification has been most studied to date, other antioxidant systems that inhibit ferroptosis, such as the CoQ oxidoreductase FSP1, are being elucidated [239]. While the exact mechanism of cell death induction by PUFA peroxidation in the context of ferroptosis has yet to be definitively uncovered, lipid peroxides might change the lipid bilayer structure, resulting in membrane permeabilization [240–242].

### Effects of p53 mutation on ferroptosis

Consistent with wild-type p53’s role in activating ferroptosis in MEFs, p53 deficiency and mutation may result in repression of ferroptosis (Fig. 5B). Common tumor-derived p53 missense mutants R175H, R273H, and R248W are unable to transcriptionally activate spermidine/spermine N1-acetyltransferase 1 (SAT1), leading to ferroptosis resistance [243]. p53-dependent SAT1 expression correlates with arachidonate 15-lipoxygenase (ALOX15) expression, which oxygenates PUFAs and induces ferroptosis [243]. Thus, tumor-derived p53 mutations protect cancer cells from ALOX15-dependent ferroptosis [243]. p53 R248W mutation also increases FOXM1 expression (previously mentioned in “Effects of p53 mutation on E2F1-dependent apoptosis”), resulting in enhanced MAPK signaling and ferroptosis resistance [244]. These studies suggest that wild-type p53 promotes ferroptosis, whereas p53 mutants are resistant.

Paradoxically, p53 loss and mutation can also sensitize cancers to ferroptosis (Fig. 5B). Wild-type p53 inhibits ferroptosis by transactivation of the phospholipase A2β (iPLA2β) [245]. iPLA2β cleaves oxidized fatty acids from the plasma membrane, enabling detoxification by antioxidant systems and suppressing ferroptosis [245]. Tumor-derived p53 mutants lose the ability to transactivate iPLA2β, rendering cells with p53 mutation more sensitive to ferroptosis [245]. Depletion of wild-type p53 in colorectal cancer (CRC) cells enhances sensitivity to ferroptosis induction by the class I FIN erastin [234]. Loss of p53 in CRC cells enhances ferroptosis by plasma membrane-bound dipeptidyl peptidase 4 (DPP4), which interacts with NOX1, enhancing lipid peroxidation [234]. Thus, p53’s relationship to ferroptosis is context-dependent and could be dependent on DPP4 abundance, which is high in CRC compared with breast cancer and osteosarcoma, where p53 represses *SLC7A11* [234]. Furthermore, tumor-derived p53 mutants enhance ferroptosis induction in CRC. Compared with p53 wild-type cells, CRC cells bearing p53 mutations were more sensitive to ferroptosis by erastin [234]. Coexpression of mutant p53 R175H in p53 wild-type CRC cells resulted in enhanced sensitivity to erastin [234]. Reconstitution of ferroptosis sensitivity in p53 wild-type cells by expression of p53 R175H might be mediated by mutant p53 protein interactions with wild-type p53. These results demonstrate that p53 mutation might serve as a biomarker for effective ferroptosis induction in CRC. However, context-dependent differences in ferroptosis sensitivity due to p53 mutation highlight the need for identification and characterization of ferroptosis modulators such as DPP4.

p53 mutation can also sensitize cells to ferroptosis by altering iron metabolism. Reconstitution of tumor-derived p53 alleles in p53-null lung adenocarcinoma cells alters induction of iron-responsive genes compared with reconstitution with wild-type p53 [246]. Furthermore, expression of p53 missense mutants sensitized cells to ferroptosis by erastin compared with wild-type p53 [247]. These findings demonstrate that the dysregulation of iron handling might contribute to p53-mutant cancers ferroptosis sensitivity [247]. p53-mutant cancers may also be susceptible to ferroptosis induction due to impaired p21 transactivation (discussed under “Effects of p53 mutation on E2F1-dependent apoptosis”). p21 transactivation induced by p53 activation with nutlin-3 impairs ferroptosis in lung adenocarcinoma and fibrosarcoma [248]. Interestingly, ferroptosis repression is not recapitulated by CDK4/6 inhibition, and the authors postulate that either alternate CDK inhibition by p21 or enhanced GSH retention by p21 could underlie ferroptosis resistance [238, 248, 249]. As discussed, tumor-derived p53 mutants are frequently impaired in their ability to transactivate p21 [93]. Cancers bearing these mutations may be particularly susceptible to ferroptosis induction given the protective role for p21.

In summary, p53 loss and mutation serve to potentiate or inhibit ferroptosis in a context-specific manner. Contextual differences could be attributed to modulators of p53 function such as DPP4 [234]. Notably, ferroptosis sensitivity is affected by p53 mutation, which can impair p53 structural stability or ability to bind DNA, induce dominant-negative activity over wild-type p53, and impart novel functions unseen in wild-type p53. The present studies suggest that ferroptosis inducers (FINs) such as erastin may be highly relevant in treating p53-mutant cancers; however, the contexts in which FINs are most effective depends on p53 status and modulators of p53 function.

### Ferroptosis in cancer therapy

Several recent reviews have been produced discussing ferroptosis in cancer therapy, reflecting substantial interest in ferroptosis induction as a general strategy for combating cancer [250–255]. p53 mutation leads to substantial dysregulation of iron metabolism, ROS, and effectors of ferroptosis. Furthermore, many cancers require high iron load to sustain increased proliferative and metabolic activity [256]. Generally, increased iron load in cancer is balanced by a concomitant increase in ROS scavenging to limit the deleterious effects of iron-induced ROS [257]. The combination of p53 mutation, high iron abundance, and increased reliance on ROS scavenging in cancers is a promising target for ferroptosis induction. Furthermore, FINs can be combined with existing chemotherapies to enhance treatment efficacy, particularly in the setting of treatment resistant cancers caused by p53 mutation. For example, cisplatin activity is enhanced by FINs, presenting an exciting combination that may surmount cisplatin resistance p53-mutant cancers [258–261]. Induction of ferroptosis has been associated with the inhibition of resistance to gemcitabine [262]. The potential role of ferroptosis in chemoresistance reversal is highly promising [252].

Due to the pleiotropic role of p53 in ferroptosis, however, applying p53 status as a biomarker for ferroptosis induction remains challenging. As mentioned above, the link between p53 status and ferroptosis sensitivity is complex and dependent on factors that are not yet fully understood. No current clinical trials applying ferroptosis inducers utilize p53 status in participation criteria. However, further elucidation of the effects of p53 mutation and cellular contexts influencing ferroptosis sensitivity may yield effective biomarker-driven trial designs that optimize FIN efficacy in cancer therapy. The clinical translation of ferroptosis induction is aided by prior FDA approval of several agents that induce ferroptosis. Sulfasalazine and other inhibitors of system xc^−^ such as riluzole and lanperisone have already received FDA approval for medical conditions other than cancer, enabling their clinical translation [235]. Statins, which are a commonly prescribed in the treatment of hyperlipidemia, bolster the ferroptotic response in cells by inhibition of CoQ10 production, an antioxidant involved in scavenging ROS [263]. Experimental FINs under preclinical investigation are emerging that bypass chemoresistance in p53-mutant settings. FINO_2_, a small molecule which induces ferroptosis by inhibiting GPX4, is effective in both p53 wild-type and mutant cell lines [264, 265]. MON-p53, an iron-coated nanoparticle bearing a p53 expression plasmid, induces substantial ferroptosis in cancer cell lines and reduced tumor burden in mouse xenograft models [266]. Such approaches for the induction of ferroptosis for the treatment of p53-mutant cancers are still in the early days of development and may provide valuable pharmaceuticals for combating p53-mutant cancers.

## Conclusions

p53 mutation has broad, often paradoxical functions in both tumor promotion and suppression. The complexity of interrelated signaling networks influenced by p53 mutation has hindered the development of effective and generally applicable cancer therapies. However, as biomedical researchers learn more about the specific effects of p53 mutations, pharmaceutical intervention targeting p53-mutant cancers is becoming more feasible. The anticancer agents we discuss in this review are demonstrably effective in inducing cell death in p53-mutant cancers. These agents and other activators of the RCD pathways we discuss may provide effective therapies for p53-mutant cancers, which are typically aggressive and associated with poor patient prognosis. However, the efficacy of these agents is highly context-dependent, and fully harnessing the power of these promising therapeutics requires detailed understanding of how p53 mutation affects chemoresistance pathways and the tumor intracellular and extracellular environments. As advances are made in patient tumor characterization and toward a complete understanding of the complex networks influenced by p53 mutation, therapies activating RCD pathways may yet pave the way for the next generation of efficacious precision medicines for p53-mutant cancers.

## Data Availability

Data sharing is not applicable to this article as no datasets were generated or analyzed during the current study.

## Abbreviations

ALOX: Arachidonate lipoxygenase
ANT: Adenine nucleotide translocator
APAF-1: Apoptotic peptidase activating factor 1
ATM: Ataxia telangiectasia mutated
ATP: Adenosine triphosphate
BAK: BCL2 antagonist/killer 1
BAX: BCL2-associated X protein
BCL2: BCL2 apoptosis regulator
CDK: Cyclin-dependent kinase
cIAP: Cellular inhibitor of apoptosis protein
CRC: Colorectal cancer
CsA: Cyclosporine A
CypD: Cyclophilin D
DBD: DNA binding domain
DIP: Alias of GRAMD4, death-inducing protein
DPP4: Dipeptidyl peptidase 4
DR: Death receptor
E2F1: E2F transcription factor 1
ECM: Extracellular matrix
EMT: Epithelial–mesenchymal transition
ER: Endoplasmic reticulum
FADD: Fas associated via death domain
FDA: US Food and Drug Association
FIN: Ferroptosis inducer
FOXM1: Forkhead box M1
GPX4: Glutathione peroxidase 4
HNSCC: Head and neck squamous cell carcinoma
IMM: Inner mitochondrial membrane
IP_3_R: Inositol 1,4,5-triphosphate receptor
LUBAC: Linear ubiquitin chain assembly complex
MDM2: Mouse double minute 2
MEF: Mouse embryonic fibroblast
MLKL: Mixed lineage kinase domain-like pseudokinase
MPT: Mitochondrial permeability transition
mPTP: Mitochondrial permeability transition pore
NOX: NADPH oxidase
NF-κB: Nuclear factor kappa B
NOXA: Alias of PMAIP1, phorbol-12-myristate-13-acetate-induced protein 1
PI3K: Phosphoinositide-3-kinase
pRB: RB transcriptional corepressor 1
PTEN: Phosphatase and tensin homolog
PUFA: Polyunsaturated fatty acid
PUMA: P53-upregulated modulator of apoptosis
RCD: Regulated cell death
RIPK1: Receptor interacting serine/threonine kinase 1
RIPK3: Receptor interacting serine/threonine kinase 3
ROS: Reactive oxygen species
RPL5: Ribosomal protein L5
RPL11: Ribosomal protein L11
rRNA: Ribosomal RNA
RRP1B: Ribosomal RNA processing 1B
SAT1: Spermidine/spermidine N1-acetyltransferase 1
SLC7A11: Solute carrier family 7 member 11
DIABLO: Diablo IAP-binding mitochondrial protein or SMAC
TNF: Tumor necrosis factor
TNFR1: Tumor necrosis factor receptor 1
TopBP1: DNA topoisomerase II binding protein 1
TRADD: TNFRSF1A associated via death domain
TRAP1: TNF receptor associated protein 1 or HSP75
VDAC: Voltage-dependent anion channel
